# Ten Years of Experience With a Telemedicine Platform Dedicated to Health Care Personnel: Implementation Report

**DOI:** 10.2196/42847

**Published:** 2024-01-26

**Authors:** Claudio Azzolini, Elias Premi, Simone Donati, Andrea Falco, Aldo Torreggiani, Francesco Sicurello, Andreina Baj, Lorenzo Azzi, Alessandro Orro, Giovanni Porta, Giovanna Azzolini, Marco Sorrentino, Paolo Melillo, Francesco Testa, Francesca Simonelli, Gianfranco Giardina, Umberto Paolucci

**Affiliations:** 1 Advisory Council of e-Health and Telemedicine University of Insubria of Varese-Como Varese Italy; 2 TM95 Srl Milan Italy; 3 Italian Association of Telemedicine and Medical Informatics Milan Italy; 4 Department of Life Sciences and Biotechnologies University of Insubria Varese-Como Italy; 5 Department of Medicine and Surgery, University of Insubria Varese-Como Italy; 6 Alfa Design Studio Milan Italy; 7 T&C Srl Milan Italy; 8 Institute of Biomedical Technologies National Research Council Milan Italy; 9 Bms Farm Law Firm Milan Italy; 10 Multidisciplinary Department of Medical, Surgical and Dental Sciences University of Campania Luigi Vanvitelli Naples Italy; 11 DDay.it Milan Italy; 12 Up Invest Srl Milan Italy

**Keywords:** telemedicine, ophthalmology, eHealth, informatics platform, health care professional, patient care, information technology, data warehouse

## Abstract

**Background:**

Telemedicine, a term that encompasses several applications and tasks, generally involves the remote management and treatment of patients by physicians. It is known as transversal telemedicine when practiced among health care professionals (HCPs).

**Objective:**

We describe the experience of implementing our telemedicine Eumeda platform for HCPs over the last 10 years.

**Methods:**

A web-based informatics platform was developed that had continuously updated hypertext created using advanced technology and the following features: security, data insertion, dedicated software for image analysis, and the ability to export data for statistical surveys. Customizable files called “modules” were designed and built for different fields of medicine, mainly in the ophthalmology subspecialty. Each module was used by HCPs with different authorization profiles.

**Implementation (Results):**

Twelve representative modules for different projects are presented in this manuscript. These modules evolved over time, with varying degrees of interconnectivity, including the participation of a number of centers in 19 cities across Italy. The number of HCP operators involved in each single module ranged from 6 to 114 (average 21.8, SD 28.5). Data related to 2574 participants were inserted across all the modules. The average percentage of completed text/image fields in the 12 modules was 65.7%. All modules were evaluated in terms of access, acceptability, and medical efficacy. In their final evaluation, the participants judged the modules to be useful and efficient for clinical use.

**Conclusions:**

Our results demonstrate the usefulness of the telemedicine platform for HCPs in terms of improved knowledge in medicine, patient care, scientific research, teaching, and the choice of therapies. It would be useful to start similar projects across various health care fields, considering that in the near future medicine as we know it will completely change.

## Introduction

### Context

Medicine has typically involved physicians engaging face to face with patients. However, many teleconsultation projects have now been developed, particularly during the COVID-19 pandemic era, which has boosted teleconsultations in all medical specialties [1-4].

Alongside telemedicine between physicians and patients, there is also transversal telemedicine, which is conducted between health care professionals (HCPs). Our experience with this topic started in 1996 and has demonstrated the feasibility of training young ophthalmic vitreoretinal surgeons working in nonoptimal environments (postwar Bosnia), using telemedicine (via a satellite link) in Milan and Sarajevo [5,6]. Input from the above experiences [7,8] constituted the basis for our understanding of the needs of HCPs and the developmental direction of the dedicated telemedicine platform, giving users access, with appropriate personal authorization, from anywhere and at any time.

### Problem Statement

The problem to be solved is the difficulty of sharing patients’ clinical data and images among health personnel for efficient evaluation. This process should be multidisciplinary, involving actors such as physicians from different specialties, nurses, technicians, orthoptists, geneticists, residents, and tutors who need access to a common database holding key patient information.

### Similar Interventions

Our scientific literature analysis identified a number of publications about implementation projects involving telemedicine platforms. These projects were mainly based on COVID-19 management and aimed to support different systems to provide health care in emergency conditions [9-11]. The purpose of these initiatives is to foster telecare and telemonitoring and to reduce the need for patients to visit hospitals or medical centers [12-17]. Our program is oriented in a different direction: the Eumeda web-based medical platform was developed for sharing patients’ medical data among physicians. The platform has expanded its services to many HCPs. This paper describes how database modules for the clinical databank and trials, as well as second opinion services, were created and have now been implemented.

## Methods

### Aims and Objectives

The aim of this implementation program was to broaden the applications of our telemedicine platform with a transversal approach targeted at health care personnel. This process took place over the last 10 years with the creation of different projects aimed at clinical data collection, teleconsultation, and gathering second opinions. Various modules have been built for the platform (Textbox 1) for use by HCPs at different times. Twelve representative modules for different clinical projects are described in Table 1 [18-23].

We identified outcome measures and evaluated overall parameters for access, acceptability, and medical efficacy of the platform (Textbox 2).

**Building a module in 8 steps. The time required for the final release varies between modules (from 1 to 3 months for more complex ones). The original source code for the modules created belongs to the medical platform.**

1. Initial agreement between the entity applying the module (university, company, institution, or representative association) and the manager of the medical platform (MP)

2. Signing of detailed operational form (with project requirements, such as the type of project, number of health care professionals and structures involved, and the importance of images) by the main users of the module and the scientific coordinator (who has knowledge of medicine planning and the potentiality and limits of medical informatics) of the MP

3. “Shoulder-to-shoulder” work by the scientific coordinator of the MP and main team programmer of the MP

4. Development of alpha software (not yet stable and still incomplete) to be shown to the entity that will use the module for changes and additions

5. Development of beta software with almost all functionalities

6. Massive data entry by the MP programmer to find bugs or software incompatibilities

7. Completion of beta software with automatic control functionalities (eg, alert icons to prevent inappropriate data from being entered, numerical limitations, and priorities to be respected in data entry or blocking of inappropriate saving) for users to check and identify any small changes required

8. Release of final version in a meeting with users, with explanatory text embedded in the module

**Table 1:** The left-hand column lists the 12 representative projects for which many modules have been built for the medical platform. The modules designed have been managed by health care personnel over the last 10 years in different locations in Italy.

Module	Description	Module type	Purpose	Timeframe of project activity	Holder	Sponsor
1	Teleconsultation in retinal diseases [18]	Second opinions^a^	Feasibility of second opinions among physicians	1 Month (during 2011)	Insubria University, Varese-Como	Comed Research nonprofit association, Milan
2	Age-related maculopathy [19]	Group^b^ (10 locations)	Acceleration of anti–vascular endothelial growth factor therapy	19 Months (2011-2012)	T&C Srl, Milan, Italy	Novartis Pharma SpA, Origgio, Italy
3	Retinal pathology samples and correlated genes [20]	Data^c^	Collection of data on gene expression	4 Months (2012-2013)	Insubria University, Varese-Como	Insubria University, Varese-Como
4	Epiretinal macular membrane [21]	Data	Collection of data on disease morphology and functionality	10 Months (2015-2016)	Insubria University, Varese-Como	Insubria University, Varese-Como
5	Inherited eye diseases	Data	Collection of data on genetic eye diseases	2017-present	Ophthalmological Unit II, University of Naples	Ophthalmological Unit II, University of Naples; Rome Foundation
6	Retinal dystrophy due to rare *RPE65* gene mutation [22]	Group (9 locations)	Collection of data on disease	16 Months (2018-2020)	Ophthalmological Unit II, University of Naples	Retina Italia nonprofit association, Milan
7	Second opinions among resident physicians	Second opinions	Feasibility of second opinions in didactics	4 Months (during 2019)	Comed Research nonprofit association, Milan	Bayer Italy SpA, Milan
8	Instrumental data in multiple sclerosis	Group (2 locations)	Collection of multidisciplinary data on disease	2019-present	Neurological Unit, Insubria, University Varese-Como	Insubria University, Varese-Como
9	Epidemiological data on COVID-19 in workers	Group (2 locations)	Search for COVID-19 in throat, saliva, and tears	3 Months (during 2020)	SEA Company, Milan Linate-Malpensa Airports	SEA Company, Milan Linate-Milan Malpensa Airports
10	SARS-CoV-2 on throat and ocular surfaces [23]	Group (2 locations)	Search for SARS-CoV-2 in throat and tears in COVID-19 patients	1 Month (during 2020)	T&C Srl, Milan. Italy	Insubria University, Varese-Como
11	Potential malignant oral lesions	Group (2 locations)	Collection of data on disease	2021-present	Orthodontics Unit, Insubria University, Varese-Como	Insubria University, Varese-Como
12	Maculopathies and anti-aging medicine	Data	Collection of data on diseases and follow-up	2022-present	Claude Boscher, MD	Claude Boscher, MD

^a^Second opinions: second opinions from health care professionals at the same or a different institution.

^b^Group: shared database used by health care professionals at more than one institution.

^c^Data: shared database used by health care professionals at a single institution.

**Result options for the questionnaire for each health care professional, with relative scores. The final score is given by the sum of the partial scores (maximum 9, minimum 3). Scores equal to or higher than 6 are considered to indicate approval.**

**Access to the network by computer or mobile devices**

- Poor: score of 1

- Good: score of 2

- Very good: score of 3

**Acceptability of the procedures**

- Poor: score of 1

- Good: score of 2

- Very good: score of 3

**Medical efficacy**

- Poor: score of 1

- Good: score of 2

- Very good: score of 3

### Blueprint Summary

#### Design of Key Features and Roadmap

The design of the implementation program was oriented to develop three types of operational modules, integrated with one another where necessary: (1) a databank of diseases for clinical or scientific studies, (2) a database for groups of HCPs in different locations, giving them access to shared data from trial studies, and (3) a functionality enabling physicians to seek second opinions. The key points of the implemented modules were easy accessibility, complete acceptability for HCPs, data reliability, and overall medical efficacy considering all health specialties. The roadmap followed these principles and several new projects involving HCPs produced specific modules, which were created for the platform and take advantage of its benefits as a whole.

#### Technological Design and Infrastructure

Since 2010, the Eumeda platform has used continuously updated versions of PHP, an HTML-embedded web scripting language built to a high standard using advanced technology [24], which has the advantage of speed, flexibility, low use of resources, and compatibility with all web servers. PHP does not require a high level of machine resources to run and is therefore very fast and lends itself to applications with external integration.

### Main Features of the Platform

Information technology services can be accessed via monitors or mobile devices and include current advanced technologies, such as the following: 24-7, 365-day-a-year access, easy data image insertion in electronic medical records, image comparison and overlapping, and SMS and email notification, when necessary, for fast interactivity.

### Customizable Modules

The platform includes customizable files called “modules” that are designed and built for each project according to its needs in collaboration with professionals from different knowledge areas (Textbox 1). Each module functions to support the features and advantages of the entire platform. No data are sent directly to or from HCPs’ hardware. HCPs are able to see data in the central database, accessing this information remotely. All HCPs have a personal access code depending on their authorization level, enabling them to view, insert, or modify data in specific fields, close the electronic medical record (EMR) data temporarily or permanently, and export data for statistical surveys. The platform allows for individual and group interaction among HCPs at different sites. A remote “prompt assistance” service is provided for each module when necessary.

### Module Functionality

**Figure 1:**
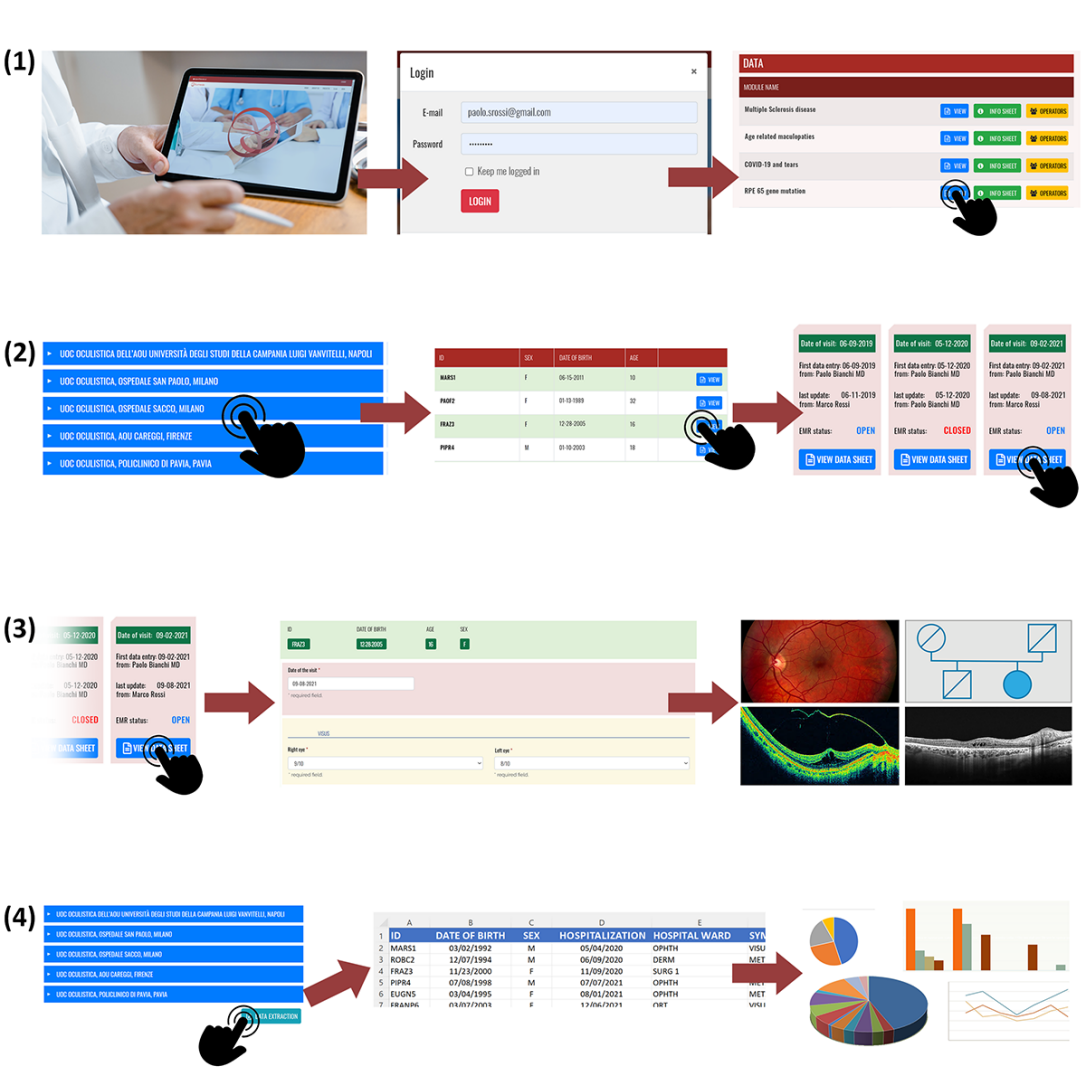
Example of the main tasks and procedures for a module (module 6 in Table 1) on the medical platform: (1) entry to the system by the health care professional with their personal access key, after which they select the modules that they are qualified and authorized to use; (2) access to a list of operative centers with their own lists of patients and respective electronic medical records relating to the first and follow-up visits; (3) individual patient electronic medical record folder, which allows for the easy and quick insertion of data and multiple images at any time, as well as access to successive masks (a repository of images is available that allows image overlapping and comparison; Figure 3); (4) quick data extraction for statistical purposes.

**Figure 2:**
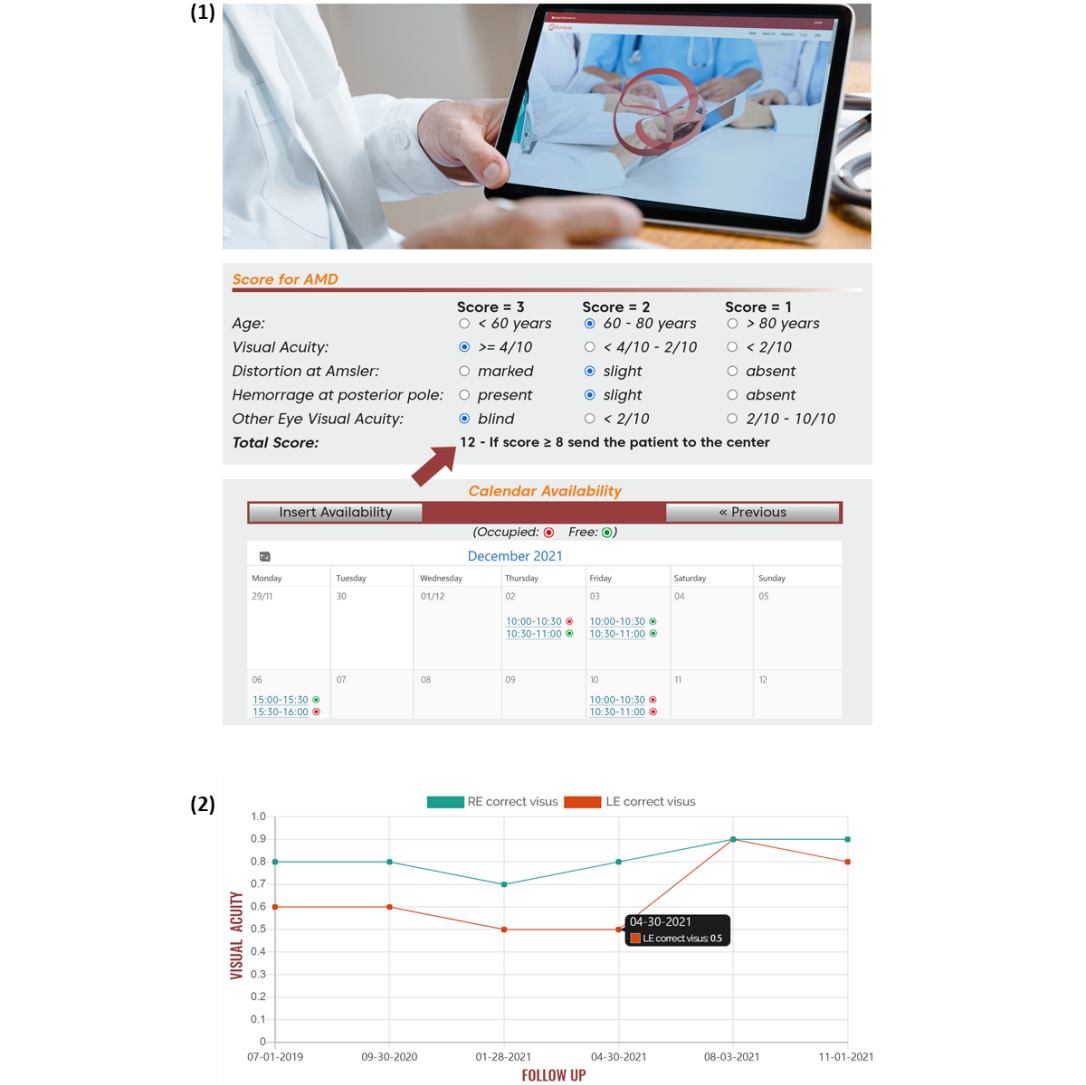
Examples of 2 special software programs designed to support health care professional activity. (1) For medical care decisions, each diagnostic variable of a disease is given a numeric value, and the software automatically provides a total score (shown by the arrow). If the value exceeds a defined score, the software advises general physicians to send the patient to an appropriate center at the next available appointment (module 2 in Table 1). (2) For tracking patients’ clinical course, visual acuity data (or any other numerical data) are inserted into a patient’s electronic medical record and the graph is updated in real time. The health care professional can see at a glance the functional course of the disease. RE: right eye; LE: left eye.

Several functions can be activated, with open pop-ups showing the rationale of each study, its population, the provenance of resources, and the operating HCPs with various authorization profiles. The data entry procedure is quick, intuitive (Figure 1), and guided by many system alerts in the case of errors. When necessary, warning notifications are sent to users via SMS or email. Special software can be created, if requested, to support HCPs’ data evaluation and clinical decisions [25-27] (Figure 2). Data extraction for statistical surveys is immediate (Figure 1). At the end of each study period, the HCPs evaluated the project using a 3-point scoring system (Textbox 2).

### Images

**Figure 3:**
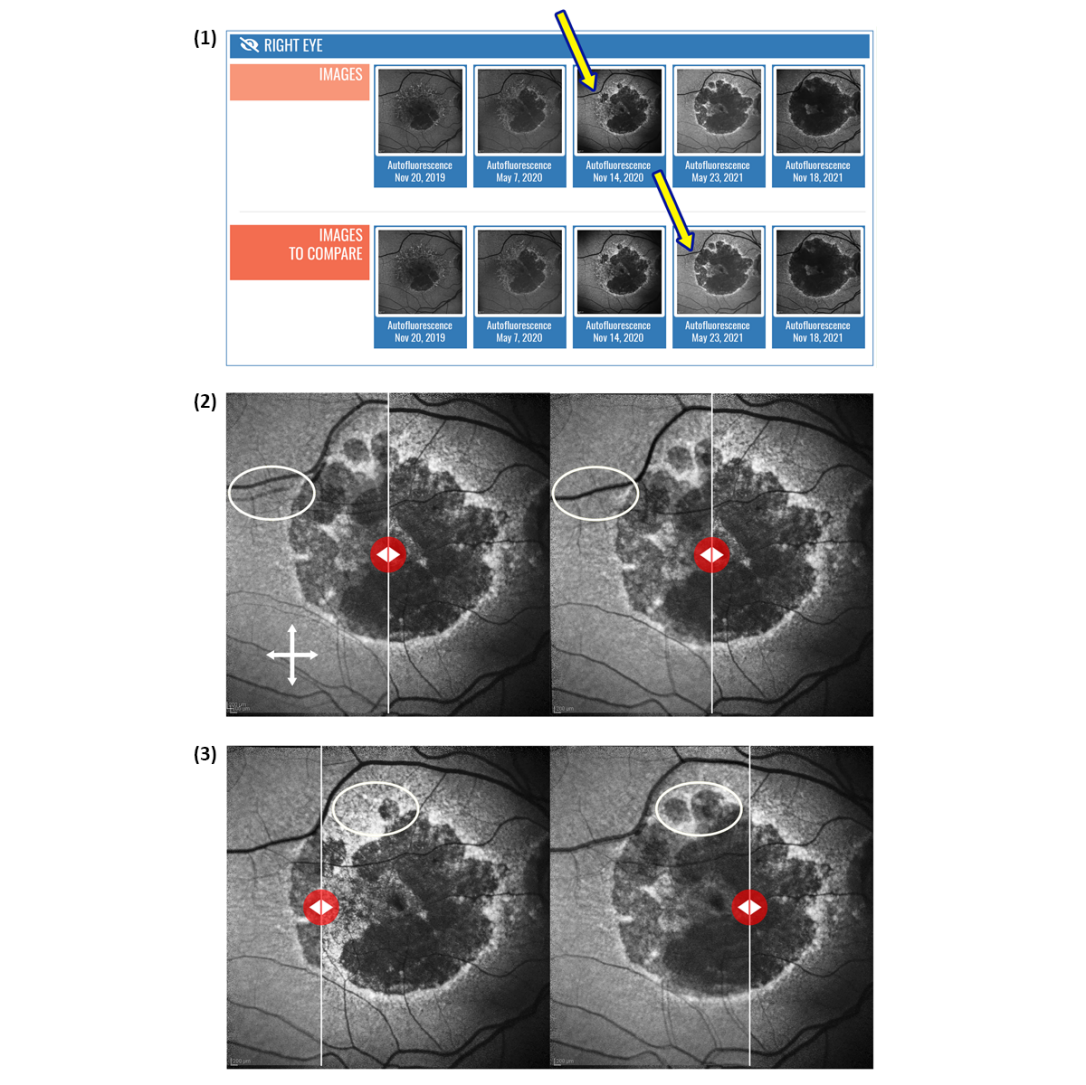
Example of image comparison: (1) selection of 2 images from a patient’s electronic medical record (in this case, the patient had degenerative retinal maculopathy) uploaded at the 6-month follow-up (shown by the arrows); (2) creation of an overlap image that can be adjusted by clicking and moving the white cross; a special transparency application allows the images to be accurately superimposed on each other (shown by the white rings); (3) evaluation of morphological changes in the disease over time (within the white rings) by moving the overlap line back and forth (shown by the white line) using the red button.

Dedicated software allows the uploading of even high-resolution images and videos in a few seconds. A shared whiteboard for all images is available for each module. Image magnification and comparison software enables morphological changes to be observed over time in detail (Figure 3).

### Type of Technology

The Eumeda software platform is closed-source and owned by a private company that grants access through contracts. The platform was developed with the Wappler (Wappler.io) integrated development environment.

#### Targets

The target user base includes physicians of different specialties, nurses, technicians, orthoptists, geneticists, residents, and tutors, all of whom rely on access to a common database holding key patient information.

**Figure 4:**
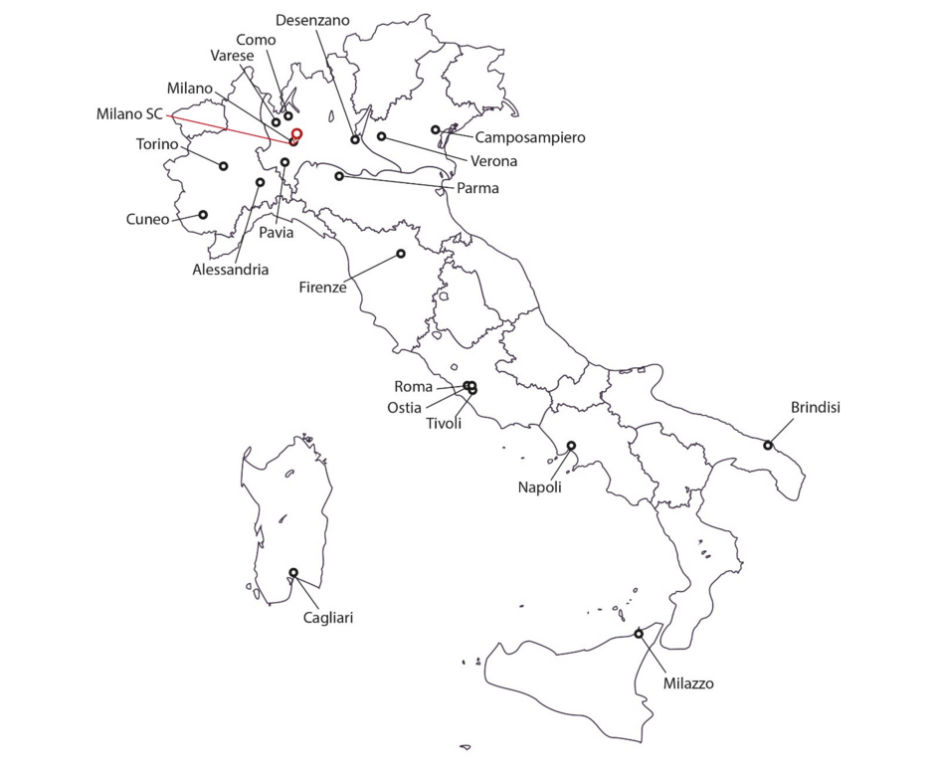
Locations of health care professionals (round dots) in Italy who have used the platform over the years (the map shows the Italian names of the cities). The Supervisory Center (SC) in Milan has responsibility for the data warehouse and help desk as well as a general coordination role.

The target sites are hospitals, private offices, and medical hub centers working with spoke-peripheral centers. We involved medical structures equipped with technology and staff prepared to use hardware and software (Figure 4).

#### Data

##### Data Location

All data were uploaded and stored in a data warehouse in Milan, Italy (Datasys Srl from 2001 to 2012; then Aruba Business Srl, provided by IRQ10 Srl, from 2013 until the present), to ensure data security and uninterrupted availability. Automated daily backups are a security measure guarding against the loss of data.

##### Data Entry Policy

HCPs must agree to the liability agreement, ownership agreement, and a code of conduct before using the platform. In all modules, data entry is performed in accordance with the guidelines of the Declaration of Helsinki and its subsequent revisions [28]. Informed consent forms are collected by the health facilities. In cases where data analysis included a therapeutic choice, approval from the relevant ethics committee or a qualified local committee was obtained, as in modules 6 and 11 in Table 1.

##### Data Security and Privacy

Data security and privacy are guaranteed by the latest generation of servers with secure backups. Data are protected on several levels: (1) individual HCPs receive access keys generated by the system; (2) subjects’ personal data are encrypted and stored in a separate table in the cloud; (3) the system binds clinical data to personal data only when accessing hardware with a special algorithm; and (4) if necessary, a ready-to-use informed consent form can be downloaded for signature.

##### Responsibility for and Ownership of Data

According to European Union (EU) and Italian rules, liability for entered medical data, including cloud storage, lies with the HCP entering such data (acting as the “controller,” as clearly defined in EU Regulation 679/16) and the manager of the medical platform and server farm (acting as “processors,” as clearly defined in EU Regulation 679/16). Ownership of the data, including the purpose and methods for processing the data, belongs to the entity applying the module, who acts as the “controller.”

#### Interoperability

The Eumeda software platform is not accessible from specific application programming interface clients by users, so it does not use data standards such as Health Level Seven. However, it implements the International Classification of Diseases, 10th revision, system internally to classify pathologies. Clinical imaging is managed with current standard protocols.

#### Participating Entities

A nonprofit organization (Comed Research) initially implemented (from 2001) the projects. Subsequently, a joint venture between 2 for-profit companies (T&C Srl and TM95 Srl) managed the platform. These partners supply hardware, create software, or participate as web designers, hosting companies, or law firms. The funders of the implementation projects are public universities, hospitals and foundations, nonprofit medical associations, private companies, and physicians working in private offices (Table 1).

The society that created the main platform is the owner of all the implementations. The entities that applied the modules hold the ownership and the intellectual property.

#### Budget Planning

The total budget covered different phases according to implementation progression and project type for a period of 1 to 3 years. The costs included preliminary planning and the final draft (up to 30%), programming for final front view on the computer screen (30%), and user training (10%), as well as the pilot phase (5%), operation (10%), initial service (10%), and ongoing reports (5%). Selected projects could be conducted for free based on their importance or visibility for the platform.

#### Sustainability

The projects were initially funded (from 2001) by a nonprofit organization (Comed Research), which relies on donations from companies or nonprofit medical associations. Since January 1, 2017, the platform has been managed by a joint venture between 2 for-profit companies. The business model is based on the type and duration of the projects developed during the implementation phase, financed by different entities. The end of the project foresees the dissemination of the results with potential permanent effects.

## Implementation (Results)

### Coverage

The projects developed during the implementation phase have national coverage, encompassing a large number of Italian regions and their referent hospitals. The developed modules evolved over time, with varying degrees of interconnectivity, in different centers in 19 cities across Italy (Figure 4). In 2 modules (modules 1 and 2 in Table 1), HCPs from the referring regional areas were closely involved. The number of HCPs (at different levels) using individual modules ranged from 6 to 114 (average 21.8, SD 28.5).

### Outcomes

Implementing the telemedicine platform allowed us to build several modules that could be used by HCPs at different times. The characteristics of 12 representative modules used over the last 10 years for different clinical projects are shown in Table 1.

Over time, our experience has led us to concentrate on three types of operational modules, integrated with one another if necessary: (1) a databank of diseases for clinical or scientific studies (eg, module 4; Table 1), (2) a database for groups of HCPs in different locations, giving them access to shared data (eg, module 6; Table 1), and (3) a functionality enabling physicians to seek second opinions (eg, module 7; Table 1).

The overall outcomes are reported in Table 2. Up to now, more than 250 HCPs have used the platform for several effective and operational projects. The total number of participants inserted in the modules is 2574. The percentage of data entered in the text or image fields for each module ranged from 20% to 95% (with an average of 65.7%). The evaluation score for each module was calculated as the sum of 3 partial scores (Textbox 2): out of all the modules, the first (module 1, the first to be created) was the one with the lowest evaluation score (Table 2). The average number of requests for technological support varied from 5 per month (in the case of simpler modules) to 9 (for more complex ones).

**Table 2:** Results pertaining to the designed modules shown in Table 1.

Module	Description	Centers involved, n	HCPs^a^ involved, n	Participants whose data were inserted, n	Text/image fields for each EMR^b^, n (fields that were filled in, %)	Beneficial effects	Evaluation score^c^ (minimum positive score)
1	Teleconsultation in retinal diseases [18]	1 Retina center, 17 territorial offices	18	52	30 (60)	Useful teleconsultation among doctors	109 (108)
2	Age-related maculopathy [19]	11 Retina centers	114	678	65 (85)	Improvements in patients’ functional final outcomes	803 (684)
3	Retinal pathology samples and correlated genes [20]	1 Ophthalmological center, 1 genetic center	11	12	65 (80)	Better understanding of molecular mechanisms	Not acquired
4	Epiretinal macular membrane [21]	2 Ophthalmologic centers, 1 human anatomy center	11	28	25 (65)	Identification of ultramicroscopic features of membranes	80 (66)
5	Inherited eye diseases^d^	1 Ophthalmological center, 1 genetic center	14	1145^e^	480^e^ (20)	Increased knowledge of genetic eye diseases	In progress
6	Retinal dystrophy due to rare *RPE65* gene mutation [22]	9 Retinal-genetic centers	28	60	260 (65)	Identification of suitable patients for therapy	200 (168)
7	Second opinions among resident physicians^d^	4 University ophthalmological departments	19	110	12 (85)	Resident physicians’ learning accelerated	140 (114)
8	Instrumental data in multiple sclerosis^d^	2 Neurological centers, 2 ophthalmological centers	6	58^e^	450^e^ (18)	Recognition of the disease in the subclinical stage	In progress
9	Epidemiological data on COVID-19 in workers^d^	2 Care offices at 2 airports	9	298	30 (90)	Collection of useful diagnostic data on COVID-19 and how the disease is transmitted	75 (54)
10	SARS-CoV-2 on throat and ocular surfaces [23]	14 Medical units	20	108	34 (95)	Increased knowledge of COVID-19	165 (120)
11	Potential malignant oral lesions	4 Medical units	6	15^e^	50^e^ (68)	Better prevention and therapy	In progress
12	Maculopathies and anti-aging medicine	1 Retina center	6	10^e^	110^e^ (58)	Significantly better care	In progress

^a^HCP: health care professional (physicians from different specialties, nurses, technicians, orthoptists, geneticists, residents, tutors [employees were excluded]).

^b^EMR: electronic medical record (for each patient, considering first visit and all follow-ups).

^c^Sum of 3 partial scores for access, acceptability, and medical efficacy at end of the active working period (described in Textbox 2).

^d^Unpublished data.

^e^At the time of writing this paper.

Clinical fallout can be identified more easily with the use of this telemedicine platform because of the visibility of a database shared by HCPs (modules 3, 4, 5, 8, and 9; Table 2). Furthermore, data on rare diseases (collected from a large number of centers) can be used to identify patients who would benefit from expensive new therapies (module 6; Table 2). By sharing medical data, physicians and residents can learn better and faster (modules 1 and 7; Table 2), and the possibility of having a databank helps them to discover potential, as yet unknown disease complications (module 10; Table 2). Patient follow-up with dedicated software helps HCPs to locate better treatment options, identify preventive interventions (modules 2 , 11 and 12; Table 2) and track patients and their outcomes in real time.

### Lessons Learned

Our program has multiple success factors that may be considered in future implementations or in the creation of similar telemedicine platforms and modules. First, the technological infrastructure of the platform is modern, highly versatile, and continuously updated by technical staff. The use of the latest generation of servers with secure, daily backups guarantees that no data loss occurs, while data security and privacy are protected on several levels, as specified in the Methods section. Second, data entry and retrieval in each module are immediate. Each module has different blocks of information that are well separated, including an explanation of the rationale of the study and practical guidance on how to insert data, as well as different HCP access profiles, patient IDs, EMRs, images, and statistical surveys. Third, no images are transmitted among HCPs. All images are stored on the main server and are viewed remotely without any deterioration. A dedicated procedure even allows the insertion of high-resolution images (through common connection links) immediately or very quickly. Rapid viewing is greatly appreciated by users, in addition to the possibility of enlarging, comparing, and superimposing, as well as being able to see in detail the morphological changes, even minimal ones, of a pathology over time (Figure 3). Lastly, different authorization profiles are given to HCPs, which enables them to access modules on the central server once they have agreed to abide by the terms of the liability and ownership agreements and the code of conduct, using personal passwords to view, change, or modify data and images. Module coordinators usually have total control of their respective modules and can compile statistical surveys using all the data, while other HCPs may only be able to enter data and images in accordance with their remit and authorized access level. A great amount of work has been undertaken to ensure that the user-friendly platform is up and running. A remote service is available by mail or telephone. All modules are visible both from monitors and mobile devices. In particular, the second opinion module may be suitable for use with mobile health (mHealth).

We consider the following points more as challenges than limits to implementation. The construction phase of each module is of critical importance, and a single medical interlocutor must be the voice of all HCPs (Textbox 1). The main mandatory factors involved in building a module include a scientific coordinator as the central figure and the participation of someone with both medical and IT skills. Finally, older HCPs tended to struggle with working on the platform, while the younger operators adapted quickly, were not disconcerted by the technology, and showed interest and satisfaction with the projects they carried out [29-34].

The presumed budgets of each project, divided into direct (eg, coordinators, IT programmers, law firms) and indirect (eg, travel, equipment, insurance) costs have been considered in the final balance.

The following recommendations may assist in overcoming many barriers to telemedicine practice among HCPs. First, the amount of preparatory work needed (Textbox 1) tends to be underestimated. Second, it is difficult to create systems for sharing text and images with appropriate levels of usability. Third, bureaucracy is often an obstacle, and self-regulation codes in telemedicine need official authorization. Fourth, a suitable “network culture” is still lacking in medicine, due to multiple technical and human factors. The success of telemedicine among HCPs requires participation, responsibility, and a desire for effective collaboration to develop knowledge for the benefit of professionals and patients.

## Discussion

### Principal Findings

The technological infrastructure of telemedicine intended strictly for HCPs is specific to this field, is not easy to implement, and must be customized for each individual project. Key persons such as scientific coordinators (with specific knowledge of medicine and IT) and program managers must be well chosen for projects to succeed. The results of our projects have shown a range of benefits, including increased medical efficacy and clinical knowledge, improved patient care, enhanced teaching and integration among hospitals, and a more effective choice of therapies. It is necessary for work to be carried out on organizational, bureaucratic, and network culture issues where these are not yet fully accepted and on sustainable business plans.

We identified some difficulties and limitations in our implementation project that may also be considered useful for future or similar telemedicine projects. Building a module in the absence of straightforward ideas forced us to make major changes during construction, meaning that the preliminary work completed had to be discarded and redone. All the software involved in the platform modules must be customized according to the needs of the HCPs, which requires time and hard work. Too many text or image fields to fill out and include in EMRs make the system difficult to use and produce a very large final database that is not fully used (as happened with module 5).

### Conclusion

In conclusion, our experience was that both physicians and patients were always satisfied to be part of this “community of health” supported by groups of HCPs working for their benefit and making them feel cared for. The detailed description of our implementation program may be useful to shorten the learning curve for others seeking to implement similar projects in many fields of medicine, which must be able to adapt to the continuously changing nature of medicine now and in the future.

## Abbreviations

EU: European Union
EMR: electronic medical record
HCP: health care professional
mHealth: mobile health

## Data Availability

The data on which this manuscript is based are available upon request to the corresponding author.
